# New genus of Ironidae (Nematoda, Enoplida) from Piip volcano (the Bering Sea)

**DOI:** 10.7717/peerj.12946

**Published:** 2022-02-16

**Authors:** Julia Zograf, Vladimir Mordukhovich

**Affiliations:** 1 A.V. Zhirmunsky National Scientific Center of Marine Biology FEB RAS, Vladivostok, Russia; 2 Far Eastern Federal University, Vladivostok, Russia

**Keywords:** Marine nematodes, Diversity, Morphology, Systematic, Ironidae, Bering sea, Piip volcano, New genus, New species, SEM

## Abstract

*Piipironus grandis* gen. et sp. nov. is described from Piip submarine volcano, the Bering Sea. *Piipironus* gen. nov. shows all main characters of Thalassironinae but differs from all known ironids in the form of the amphid (spiral *vs* pocket-like) and the simultaneous presence of precloacal papilliform supplements and tubular postcloacal organs. Pared tubular postcloacal organs have never been described before for the family Ironidae. The combination of papilliform precloacal supplements and the pair of tubular postcloacal organs described for *Piipironus grandis* gen. et sp. nov. is unique among nematodes. The study of the nematofauna of the Piip submarine volcano began quite recently, and *Piipironus* is the second new genus of nematodes described from here from one sample of bottom sediments. This can be taken as one of the examples of the hyper-high diversity of marine nematodes.

## Introduction

Members of the family Ironidae de Man, 1876 are widespread and found in various marine, brackish, and freshwater habitats. In some communities (shallow sandy sediments and mangrove mudflats in particular), ironids can be highly diverse and abundant (*e.g*. Chen & Guo, 2015; Nguyen & Gagarin, 2015). There have been several revisions of Ironidae (Andrássy, 1968; Lorenzen, 1981; Platonova & Mokievsky, 1994); however, the taxonomy of the Ironidae is still questionable. Lorenzen (1981) established the holophyly of the Ironidae based on the complex of features of the construction and mode of function of the buccal cavity structures. Currently, there are eight genera in this family belonging to two subfamilies (Smol, Muthumbi & Sharma, 2014): Ironinae de Man, 1876 (with only genus *Ironus* Bastian, 1865) and Thalassironinae Andrássy, 1976 (with seven genera, *Conilia* Gerlach, 1956, *Dolicholaimus* de Man, 1888, *Ironella* Cobb, 1920, *Parironus* Micoletzky, 1930, *Pheronous* Inglis, 1966, *Thalassironus* de Man, 1889, and *Trissonchulus* Cobb, 1920). Species of Ironinae are presumably limnetic and species of Thalassironinae are marine with exception of terrestrial *Trissonchulus baldwini* Tahseen & Mehdi, 2009. Unfortunately, *T. baldwini* is described only on females without information on the presence and structure of metanemes and without genetic data. In our opinion, *T. baldwini* has a significant similarity with *Ironus* and should be transferred to this genus.

The main diagnostic characters to distinguish genera of the family are presence/absence and type of metanemes, position of pharyngeal glands and cervical pore, presence/absence and type of caudal glands, position and number of male and female gonads, the presence/absence and type of anterior sensilla, the structure of pharynx, the shape of tail (Lorenzen, 1981; Platonova & Mokievsky, 1994; Smol, Muthumbi & Sharma, 2014). The phylogenetic analysis of 18S rDNA and 28S rDNA revealed the monophyly of Ironidae within Enoplida (Meldal et al., 2007; Bik et al., 2010; Mordukhovich et al., 2019).

As mentioned above, ironids are widespread and, in addition to shallow-water ecosystems, often inhabit deep-sea ones. In particular, the species of the genera *Parironus*, *Thalassironus*, *Trissonchulus* were described from bottom sediments below 200 m. They are also regularly found in deep-sea communities of the NW Pacific (personal observations), but to date, only one species has been described from there – *Parironus lukini* Platonova, 1984 (the Sea of Japan, 12–300 m).

In the last decade, intensive work has been carried out to study the deep-sea nematofauna of the NW Pacific (Mordukhovich & Fadeeva, 2020) including deep sea hydrothermal vent communities (Mordukhovich et al., 2020). At present time such communities have been discovered in all oceans, hundreds of regions with deep-sea hydrothermal vents and thousands of cold seeps are known (Beaulieu et al., 2013; Beaulieu, Baker & German, 2015; German et al., 2011). At macrobenthic level the deep-sea communities of vents and seeps are characterized with high number and biomass of few specialized often obligate species (Galkin, 2016; Levin et al., 2016). Meiobenthic studies of hydrothermal vent communities began relatively recently (Giere, 2009). Investigations of taxonomic composition of nematofauna in deep-sea vent ecosystems of the Pacific Ocean are mainly confined to the East Pacific Uplift (Dinet, Grassle & Tunnicliffe, 1988; Flint et al., 2006; Zekely et al., 2006; Copley et al., 2007; Gollner, Miljutina & Bright, 2013) and are still rare. In the present study, a new free-living nematode genus and species *Piipironus grandis* gen. et sp. nov. (Nematoda, Ironidae) is described from the Piip submarine volcano (the Bering Sea).

## Materials and Methods

Sediment samples were collected from the South Summit of the Piip volcano during cruise 82 of the R/V Akademik M.A. Lavrentyev from June to July 2018. Sample collection was carried out using the remotely operated vehicle (ROV) Comanche-18. On deck, the sediment was carefully sieved through 1,000, 500 and 32 μm mesh sizes and fixed with formalin (5% final concentration) in filtered seawater. In the laboratory fixed samples were sorted using stereomicroscopes. Nematodes were picked out and transferred to glycerine using the Seinhorst’s (1959) rapid method as modified by De Grisse (1969), and mounted on permanent slides. Drawings and DIC (differential interference contrast) photographs were made on an optical microscope Olympus BX 53 with the aid of a drawing tube and a digital camera respectively.

For the scanning electron microscopy, specimens were gradually dehydrated in a series of baths of increasing ethanol content, dried in a critical-point dryer, sputter-coated with gold and observed and imaged with a Ziess Sigma 300 VP scanning electron microscope (SEM) (Zograf et al., 2021).

The electronic version of this article in Portable Document Format (PDF) will represent a published work according to the International Commission on Zoological Nomenclature (ICZN), and hence the new names contained in the electronic version are effectively published under that Code from the electronic edition alone. This published work and the nomenclatural acts it contains have been registered in ZooBank, the online registration system for the ICZN. The ZooBank LSIDs (Life Science Identifiers) can be resolved and the associated information viewed through any standard web browser by appending the LSID to the prefix http://zoobank.org/. The LSID for this publication is: urn:lsid:zoobank.org:pub:FE5905E2-0338-4177-836C-2E9A311F3F0A. The online version of this work is archived and available from the following digital repositories: PeerJ, PubMed Central SCIE and CLOCKSS.

## Results

**Taxonomy**

**Order Enoplida Filipjev, 1929**

**Family Ironidae de Man, 1876**

**Diagnosis** (after Lorenzen, 1981, 1994; Platonova & Mokievsky, 1994; Smol, Muthumbi & Sharma, 2014, emended). Enoplida. Cuticle smooth or with fine striation. Mouth with three or six lips, head often set off. Anterior sensilla in three circles. Different combinations are possible: all setiform, all papilliform, only inner labial papilliform, or only cephalic sensilla setiform. Metanemes evident or not. Buccal cavity elongated armed at the anterior edge with 3–5 movable claw-like teeth which can be bifurcated. Denticles may be present at the anterior edge. In juveniles, the replacement teeth positioned in pharyngeal pouches behind the functional ones. Pharyngeal glands do not open through the teeth, but further back in the buccal cavity. The pharynx inserts, at least in some genera, into the body cuticle in the buccal cavity region. Females didelphic-amphidelphic with antidromously reflexed ovaries, rarely monodelphic-opisthodelphic. Males diorchic with opposed testes or a single anterior testis. Papilliform and/or tubular supplementary copulatory organs may be present. Tail conico-cylindrical, mostly long and thread-like at its end, sometimes conical or wide and rounded. Caudal glands present or absent.

List of valid genera (Smol, Muthumbi & Sharma, 2014):

*Ironu*s Bastian, 1865

*Conilia* Gerlach, 1956

*Dolicholaimus* de Man, 1888

*Ironella* Cobb, 1920

*Parironus* Micoletzky, 1930

*Pheronous* Inglis, 1966

*Thalassironus* de Man, 1889

*Trissonchulus* Cobb, 1920

**Genus *Piipironus* gen. nov.**

urn:lsid:zoobank.org:act:63E896E5-3A53-42F5-8321-6B842FF4CED1

**Genus description.** Ironidae. Cuticle finely striated. Buccal cavity consisting of two parts: spacious anterior part with three big solid teeth (one dorsal and two lateroventral) and small denticles and long narrow posterior part with thick tubular cuticularized walls. Three lips. Labial and cephalic sensilla setiform in three separate circles, outer labial sensilla the largest. Amphid unispiral. Females didelphic, amphidelphic with antidromously reflexed ovaries. Males with two opposed outstretched testes. Pre- and postcloacal supplementary structures may be present. Tail short, conical, caudal glands absent.

**Etymology.** Genetic name is a composite of prefix Piip- (refereeing to the type location Piip volcano) and generic name Ironus referring to relation to Ironidae family. Masculine in gender.

**Differential diagnosis.** The new genus differs from all known ironids in the form of the amphid (spiral *vs* pocket-like) and the presence of postcloacal tubular organs. *Piipironus* gen. nov. is most similar to genus *Ironella* in the setiform labial and cephalic sensilla situated in three circles. In addition to the features of the structure of the amphid and supplementary organs, described genus differs from *Ironella* by the absence of caudal glands and by the shape of tail (short blunt *vs* conico-cylindrical).

**Type species. *Piipironus grandis* gen. et sp. nov.**

**Figure 1:**
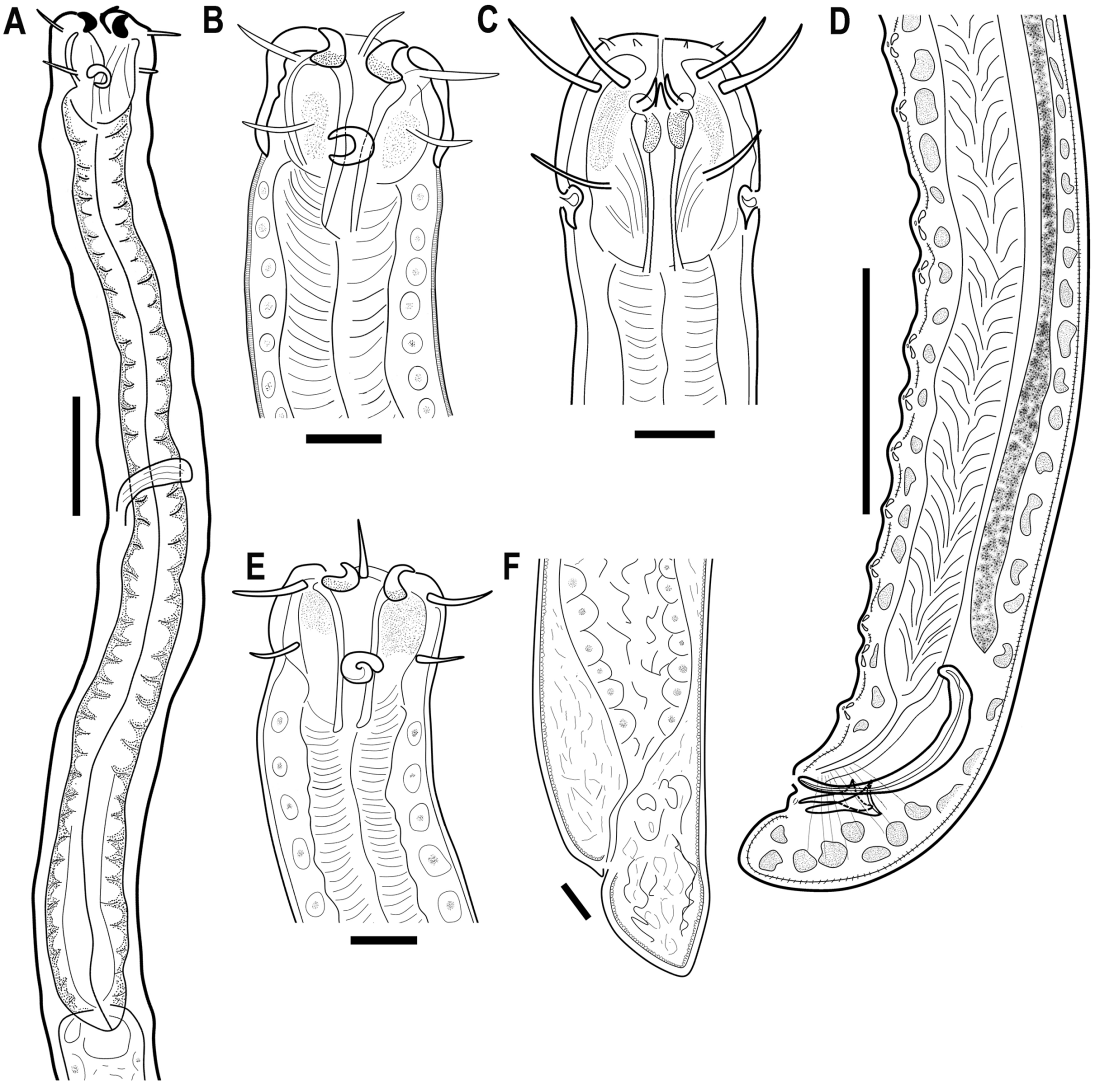
*Piipironus grandis* gen. et. sp. nov. (A) Anterior end of the male, latral view. (B) Head of the male, lateral view. (C) Head of the male, dorsal view. (D) Posterior end of the male with copulatory apparatus. (E) Anterior end of the female, lateral view. (F) Posterior end of the female. Scale bars: A, D = 50 µm; B, C, E, F = 20 µm.

**Figure 2:**
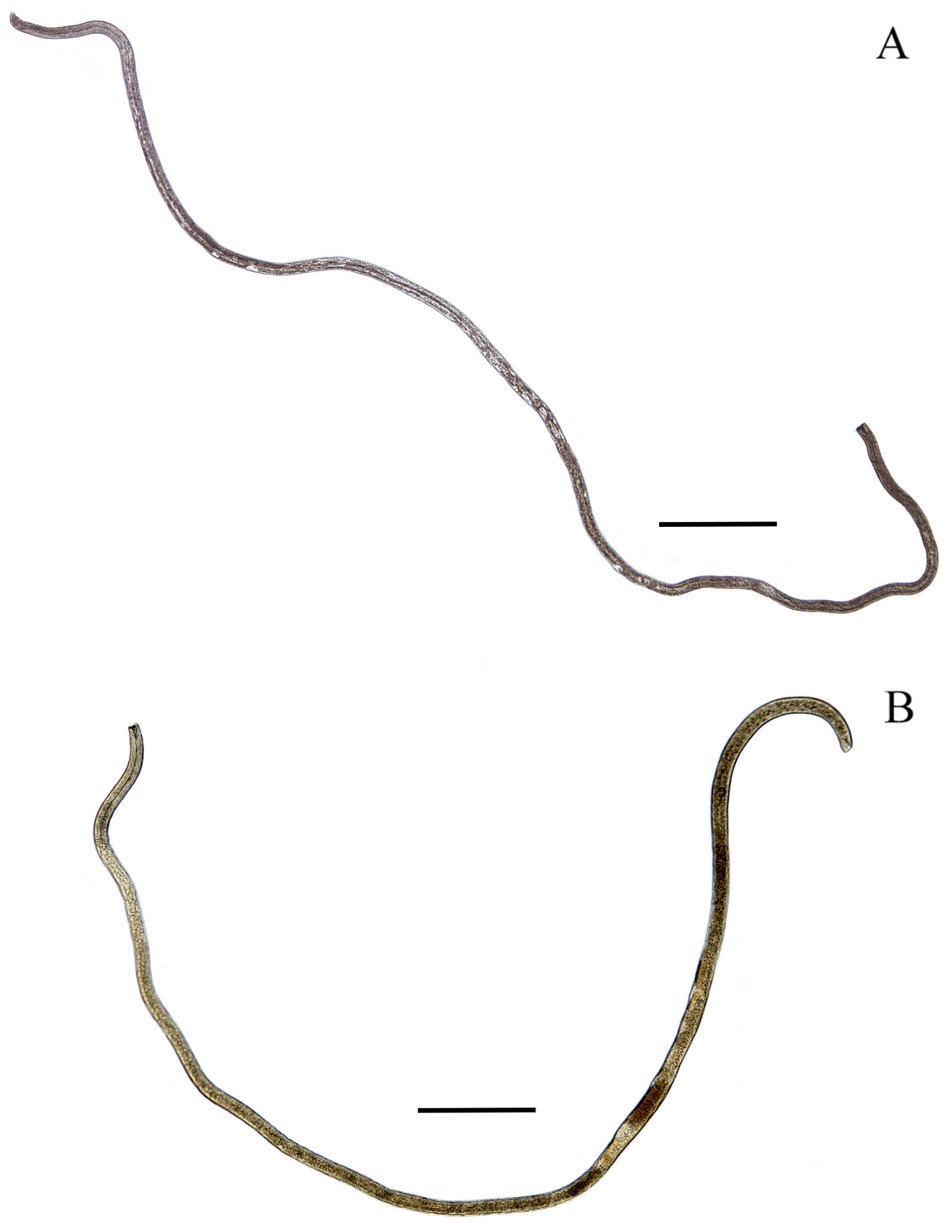
*Piipironus grandis* gen. et. sp. nov. Light microscopy. (A) Male holotype, entire body. (B) Female paratype, entire body. Scale bars: 500 µm.

**Figure 3:**
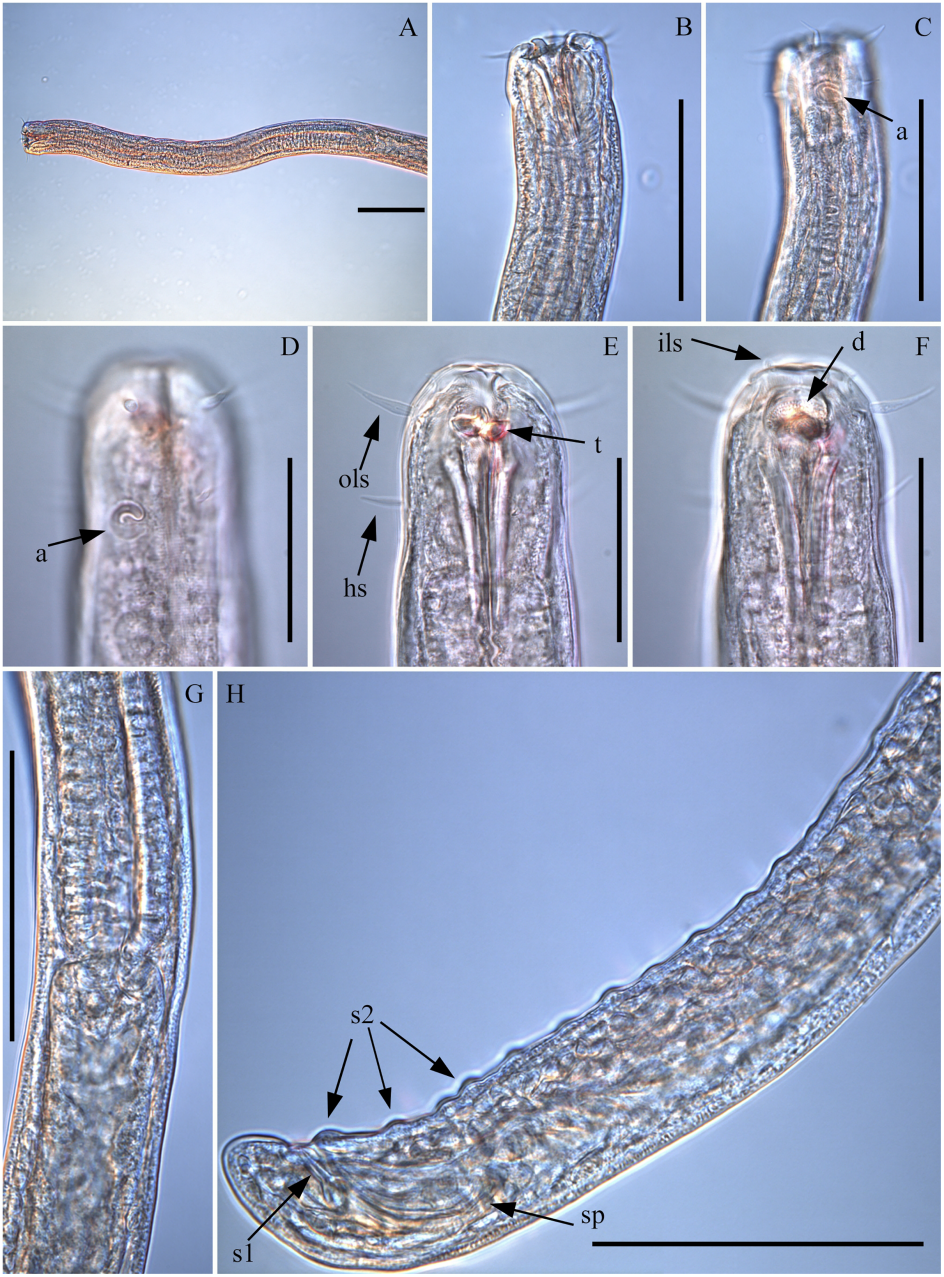
*Piipironus grandis* gen. et. sp. nov. Light microscopy. (A) Anterior end of the male. (B) Head end of the male, lateral view. (C) Head end of the male showing amphid (a). (D) Head end of the male, lateral view. (E) Anterior end of the male showing outer labial setae (ols), head setae (hs) and teeth (t). (F) Anterior end of the male showing inner labial setae (ils) and denticles (d). Pharynx-intestine connection of the male. H. Posterior end of the male with spicules (sp), papilliform precloacal supplements (s2) and tubular postcoacal (s1) organs. Scale bars: A, B, C, G, H = 100 µm; D–F = 50 µm.

**Figure 4:**
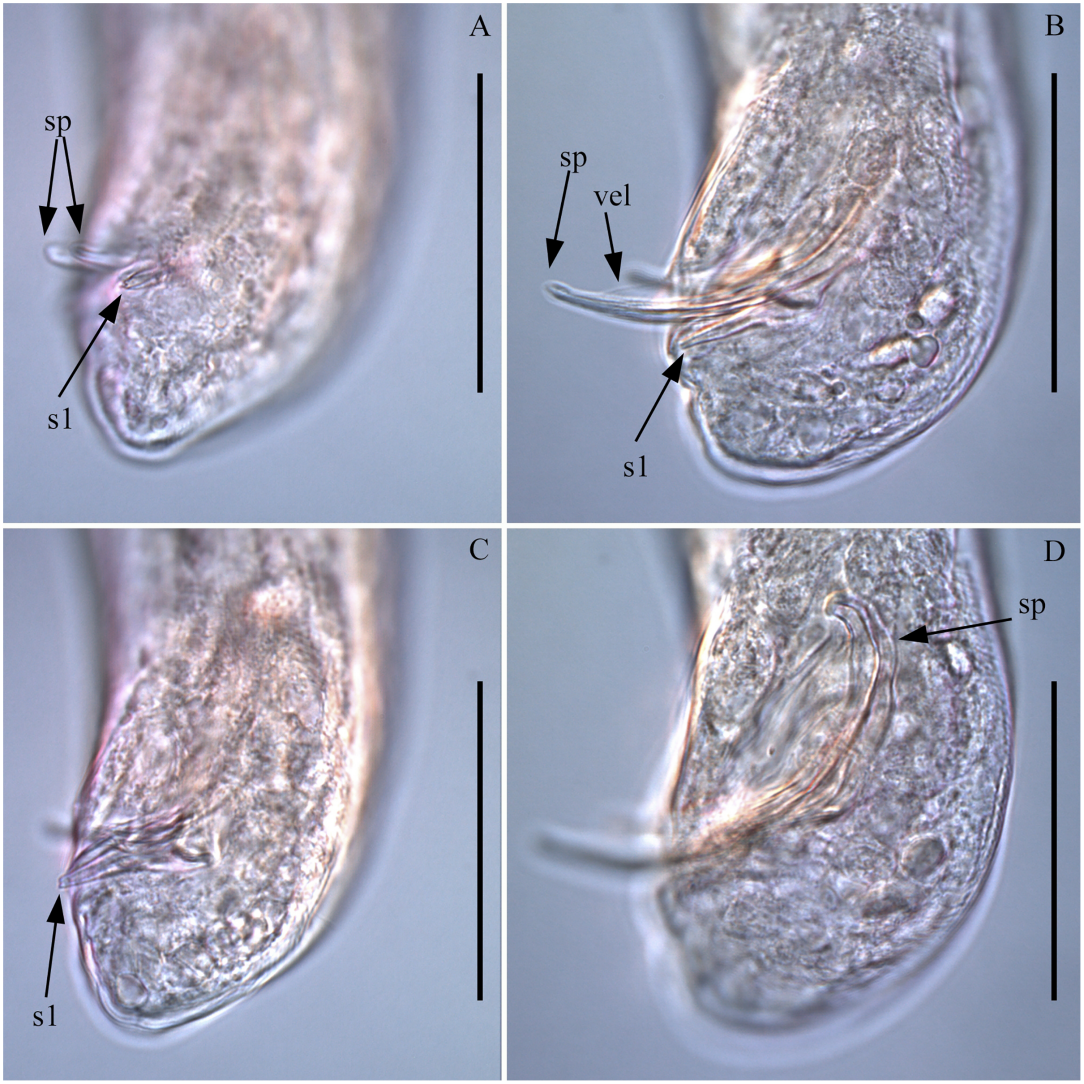
*Piipironus grandis* gen. et. sp. nov. Tail end of male. Light microscopy. (A) Tubular postcloacal organs (s1). (B) Spicules (sp) with velum (vel) protruding from cloaca. (C) Tubular postcolacal organs (s1) protruding from opening. (D) Spicules (sp) with longitudinal elevation on lateral side. Scale bars: 50 µm.

**Figure 5:**
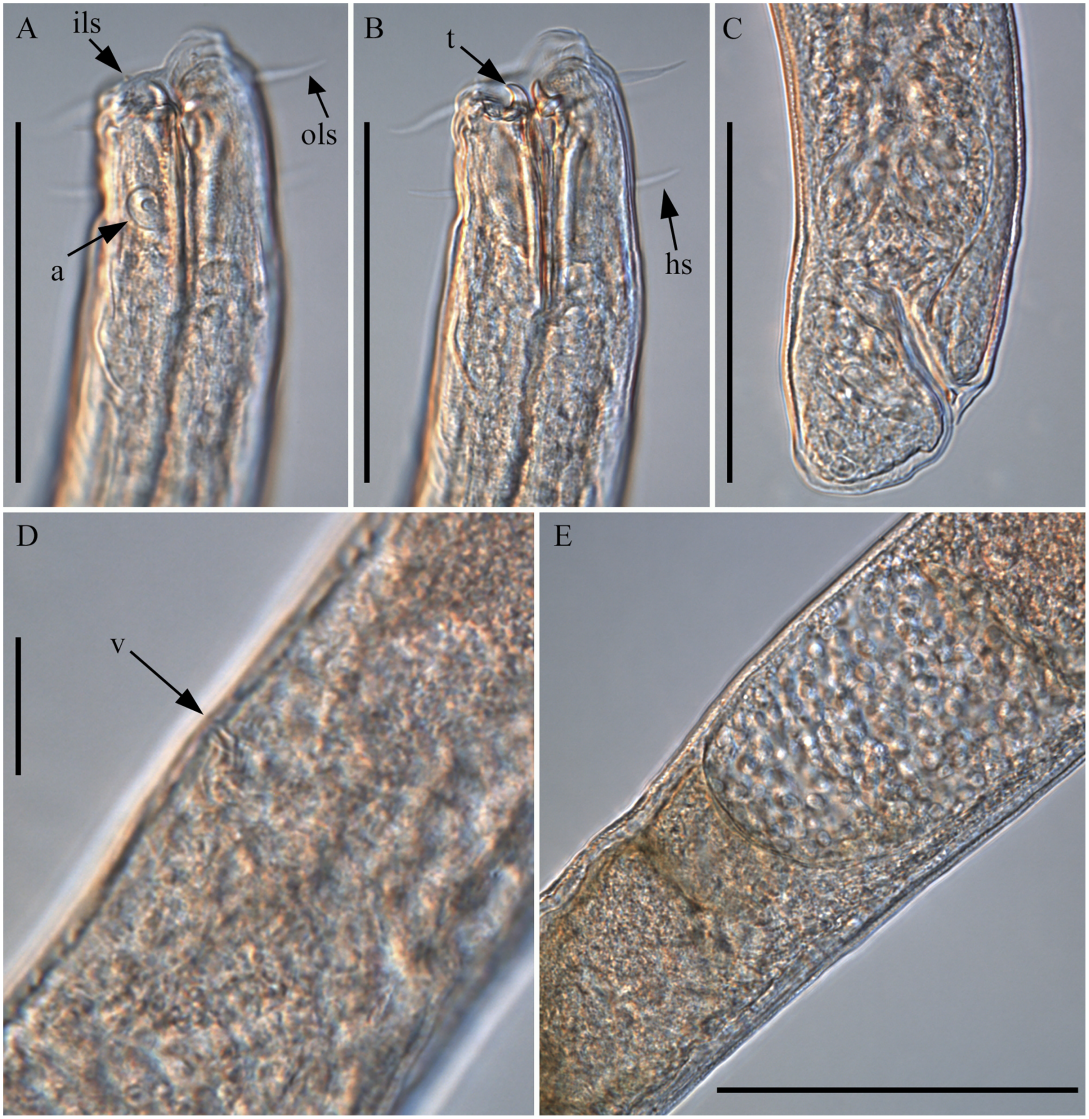
*Piipironus grandis* gen. et. sp. nov. Female patratype. Light microscopy. (A) Anterior end showing inner (ils) and outer (ols) labial setae and amphid (a). (B) Anterior end showing teeth (t) and head seate (hs). (C) Posterior end. (D) Vulvar opening (v). (E) Uterus filled with spermatozoa. Scale bars: A, B, C, E = 100 µm, D = 25 µm.

**Figure 6:**
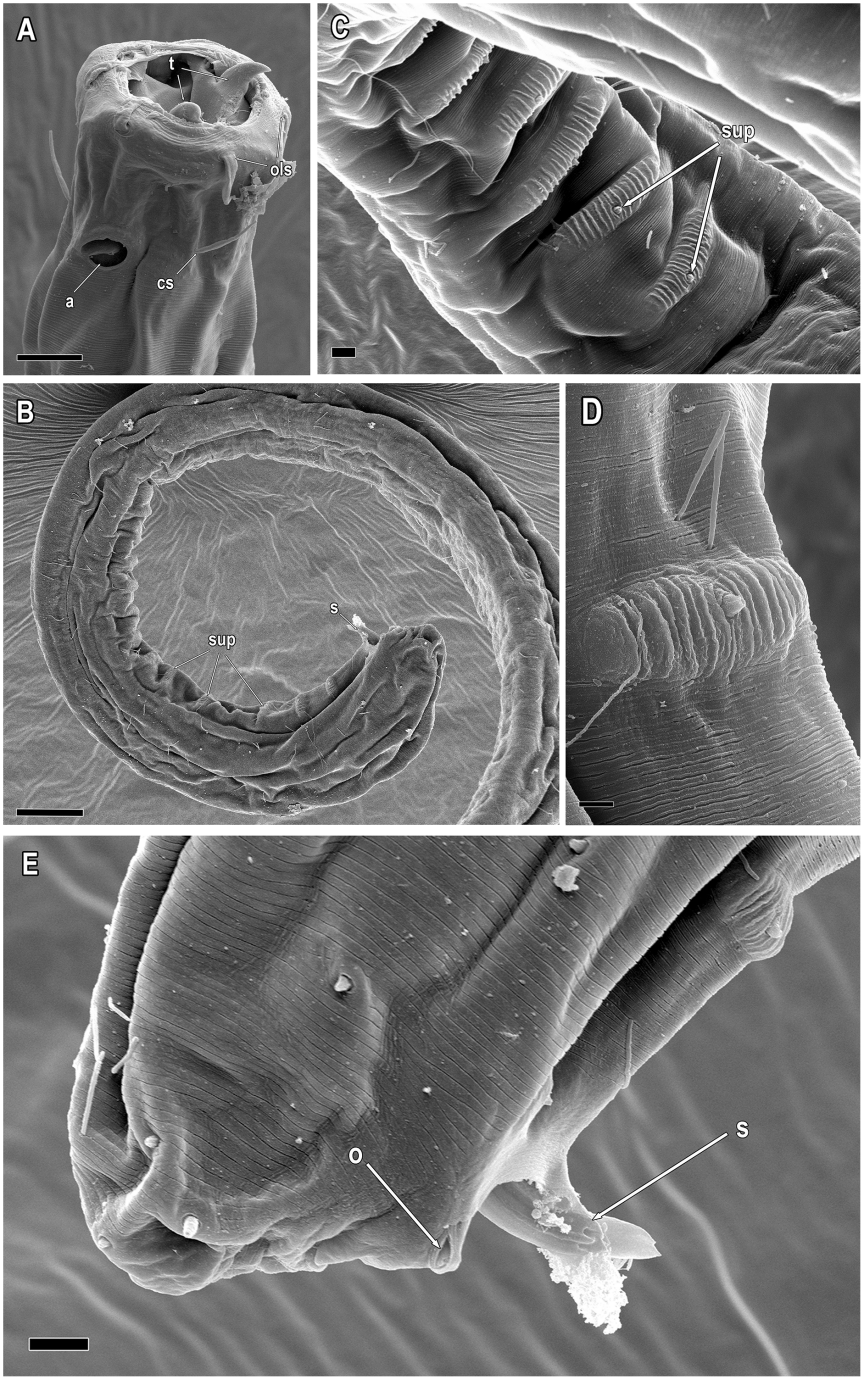
*Piipironus grandis* gen. et. sp. nov. Male paratype. Scanning electron microscopy. (A) Anterior end showing outer labial (ols) and head setae (cs), amphid (a) and teeth (t). (B) Posterior end showing precloacal supplements (sup) and spicules (s). (C) Papilliform precolacal supplements (sup). (D) Papilliform precoacal supplement on the cuticular wrinkled rising. (E) Tail with spicules (s) and opening of postcoacal tubular organ (o). Scale bars: A = 10 µm; B = 20 µm; C = 2 µm; D = 1 µm; E = 3 µm.

**Figure 7:**
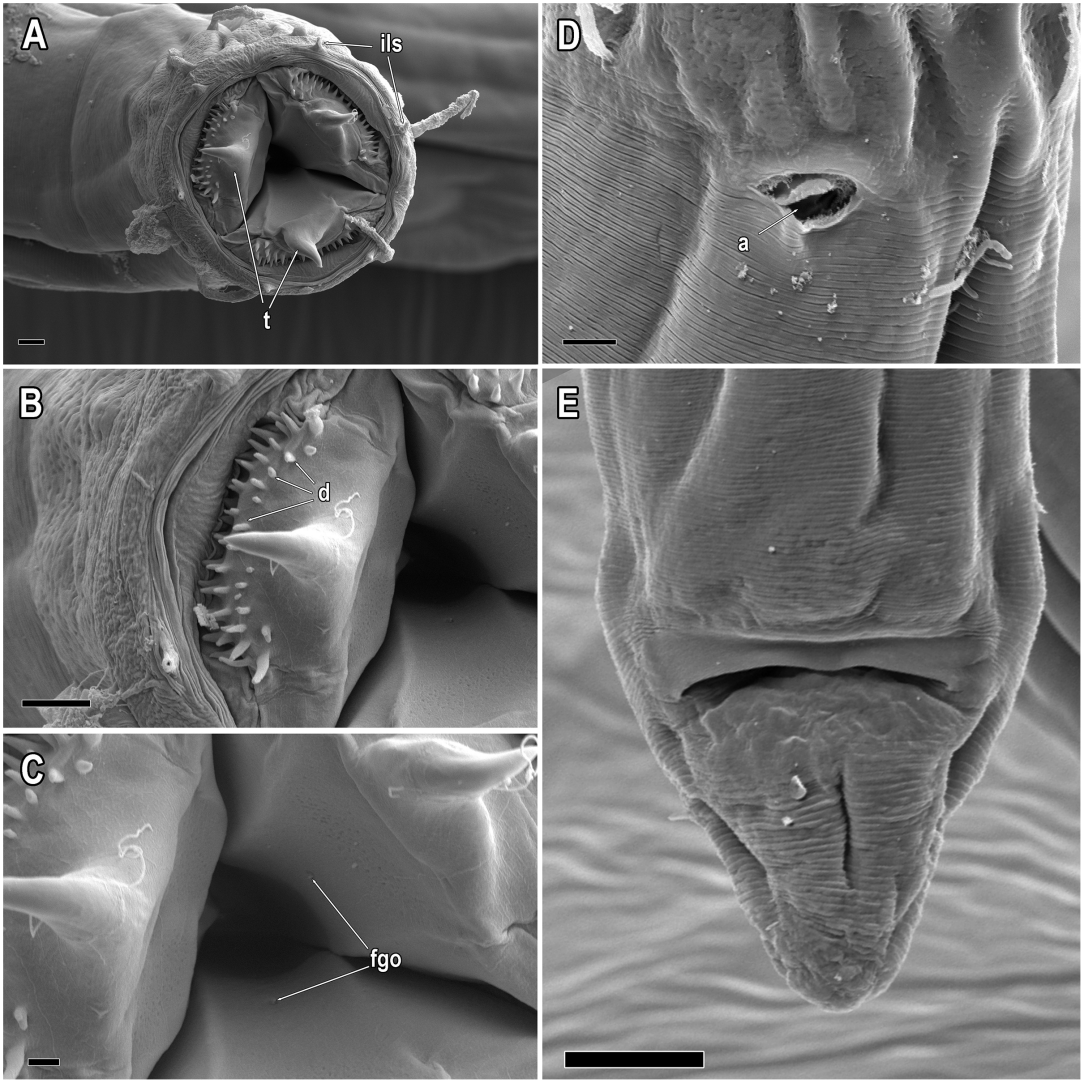
*Piipironus grandis* gen. et. sp. nov. Female paratype. Scanning electron microscopy. (A) Anterior end, apical view. Three teeth (t) protruding from mouth opening. Lips with inner labial setae (ils). (B) Tooth with denticles (d). (C) Faringeal glands opening (fgo). (D) Amphideal fovea (a). (E) Tail end, ventral view. Scale bars: A, B, D = 3 µm; C = 1 µm; E = 10 µm.

***Piipironus grandis* gen. et sp. nov.**

(Figs. 1–7; Table 1)

**Table 1:** Morphometrics of *Piipironus piipironus* sp. nov. (all measurements are given in μm unless dimensionless).

Character	Holotype male	Paratype males (*n* = 2)		Paratype females (*n* = 2)	
		min	max	min	max
L	6,159	5,612	6,773	6,244	7,088
V				4,156	4,743
M	58	60	63	65	69
ph. L.	522	548	598	570	641
a.b.d.	31	45	47	43	51
diam. c.s.	53	42	49	48	49
l. tail	21	22	27	17	30
l.c.s.	15	17	19	19	20
h.c.	10	11	13	13	14
amph. dist.	18	24	30	21	25
amph. W.	12	10	12	10	10
ph.b.d.	53	50	59	48	62
spic. Arch	71	65	73		
gub. L.	16	16	16		
Tooth length	12	10	11	11	12
a	106.2	89.1	109.2	90.5	109
b	11.8	10.2	11.7	11.1	10.9
c	293.3	243.5	282.9	208.1	416.9
c’	0.68	0.48	1	0.39	0.59
S’	2.3	1.5	2.48		
V%				66.5	66.9

urn:lsid:zoobank.org:act:A915931B-BF7D-4951-AD72-DA2F3ED8A481

**Type material.** All specimens are deposited in the Museum of A.V. Zhirmunsky National Scientific Center of Marine Biology FEB RAS (Vladivostok, Russia). Holotype 1♂ (MIMB 42849). Paratypes 4♂♂ and 2 ♀♀ (MIMB 42850-42852).

**Other material.** Au-coated SEM specimens.

**Etymology.** The species name is derived from the Latin grandis (= great, big) and refers to the giant size of this nematode.

**Type locality.** Sandy sediments at the South Summit of the Piip volcano in the Bering Sea (55.382° N, 167.261° E), water depth 470 m.

**Measurements.** See Table.

**Description.** Male. Body long, cylindrical (Figs. 1 and 2). Cuticle thick with fine striation (excluding the head region), vaguely visible under the light microscope. Mouth opening surrounded with three well developed lips (Figs. 1 and 7A). Inner labial sensilla setiform. Outer labial and cephalic sensilla setiform in two separate circles (Figs. 1, 3B and 6A). Outer labial setae 15–20 µm long. Cephalic setae 10–13 µm long situated 18–21 µm from anterior end. Head not set off by a constriction. Amphideal fovea unispiral, coiled dorsally, 10–13 µm in diam., just below cephalic setae (Figs. 1, 3B, 3C and 6A). No metanemes found. Buccal cavity consists of two parts: spacious armed anterior part and long and narrow posterior part with thick tubular cuticularized walls. Buccal armature consists of three equal (one dorsal and two ventro-lateral) protrusible strong solid teeth (Figs. 1, 3E and 6A). Each tooth consists of wide base and one claw-like curved process (Figs. 1, 6A, 7A and 7B). Each tooth flanked from outer side by few irregular rows of minute denticles (Fig. 7B). Pharynx has no basal swelling. Nerve ring situated 175–219 µm (30–40% of pharynx length) from anterior end. Ventral gland and excretory pore not observed. Cardia embedded in the intestine and round. Testes diorchic, outstretched situated to the left of intestine. Spicules with velum, strong, arcuate, 1.5–2.5 a.b.d. long with longitudinal elevation on lateral sides (Figs. 1, 4A, 4B, 4D and 6E). Gubernaculum not found. 13–16 precloacal supplementary papilla, each situated at the cuticular wrinkled rising (Figs. 1, 3H, 6B–6D). Couple of postcloacal tubular organs in shape of arrowhead (Figs. 1, 4C and 6E). Tail short, blunt. Spinneret not found.

Female. Similar with male (Figs. 1, 2B, 5 and 7). Reproductive system didelphic, amphidelphic, antidromously reflected, situated to the left of intestine. Vulval opening transverse slit, slightly shifted to posterior end, situated 66–67% of total body length (Figs. 5D and 5E). Vagina cuticularised, uterus filled with small round spermatozoa 2–3 µm in diam.

**Diagnosis.** *Piipironus*. Body length 5,612–7,088 µm. Cuticle smooth under light microscope. Head not set off. Cephalic sensilla setiform. Buccal cavity 61–70 µm long, with tree equal teeth. Spicules arcuate, 67–73 µm long. 13–16 precloacal supplementary papillae. Couple of postcloacal tubular organs. Tail short, blunt, c’ 1.5–2.5.

## Discussion

Family Ironidae was established by de Man in 1876 and to date includes around 80 valid species belonging to eight genera combined into two subfamilies: Ironinae and Thalassironinae (Smol, Muthumbi & Sharma, 2014; Bezerra et al., 2021). Lorenzen (1981, 1994) established the holophyly of the Ironinae by the two holapomorphies: the delicately built, dorsolateral and ventrolateral orthometanemes occur in a strictly alternating sequence; the Ironinae are limnetic, whereas all other Ironidae are marine. For Thalassironinae holophyly has not yet been established. At the same time, metanemes is not always possible to detect, including several species of the genus *Ironus*. Representatives of many nematode genera have a very wide distribution and can be found in both freshwater and marine communities. For example, species of the genus *Oncholaimus* (Oncholaimidae) are mostly marine, but some may be found in fresh waters and terrestrial habitats (Smol. & Coomans, 2006). *Piipironus grandis* gen. et sp. nov. is marine species without metanemes so we attribute the new genus to the subfamily Thalassironinae. This assignment requires further verification, for example, using molecular-genetic data. Unfortunately, we were not able to extract DNA from our samples.

**Figure 8:**
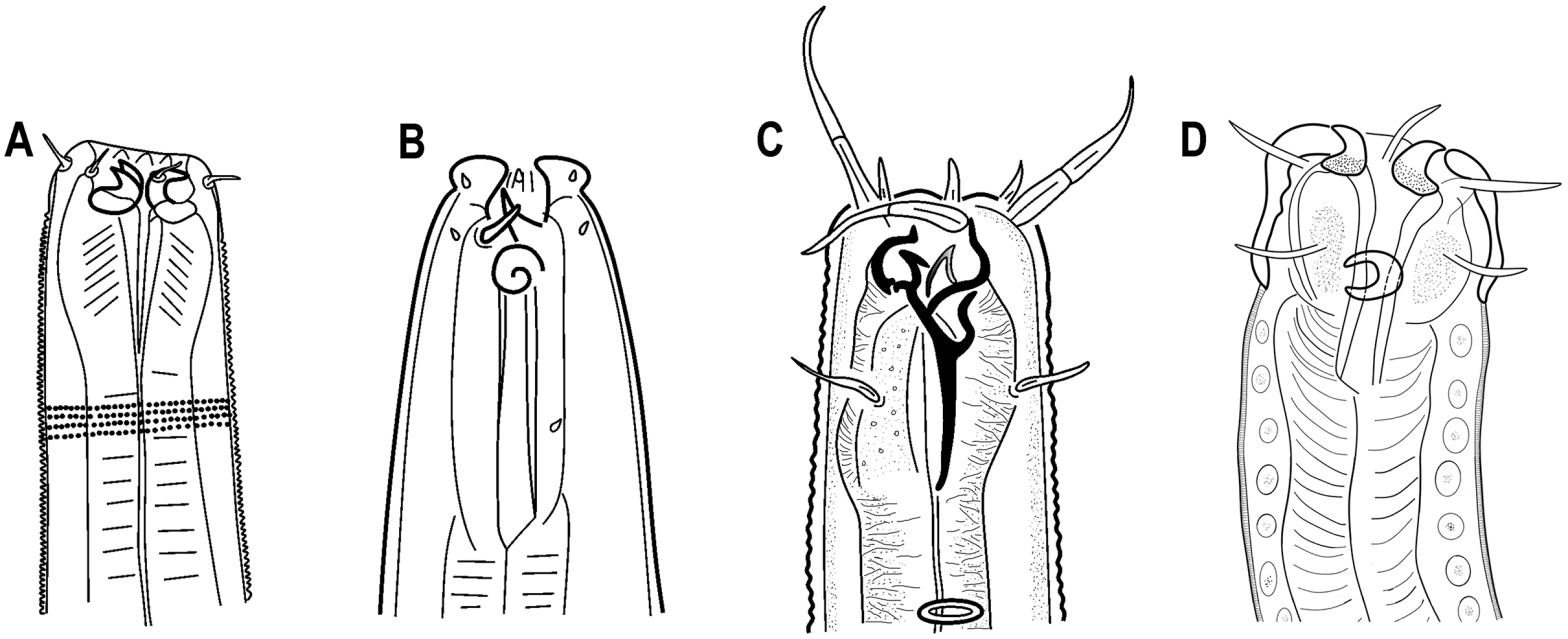
Free-living nematodes with movable hook-shaped teeth. (A) *Harpagonchus averinceri* Patonova & Potin, 1972. (B) *Paraethmolaimus appendixocaudatus* Jensen, 1994. (C) *Stenonchulus troglodytes* Schneider, 1940. (D) *Piipironus grandis* gen. et sp. nov.

Although genus *Piipironus* are characterized by the main characters of the family Ironidae, such as anterior sensilla in three circles, buccal cavity with three movable teeth at the anterior end, some features greatly differ this genus from other ironids. Unusual shape of amphid (unispiral instead of pocket-shaped in most enoplids) and finely striated cuticle at first misled us in identification as far as such a shape of amphid is usually characteristic of class Chromadoria. Specific armature of buccal cavity with movable hook-shaped teeth at the anterior edge has been described not only for ironid nematodes. Similar armament was described for nematodes belonging to different orders of nematodes: the subfamilies Harpagonchinae Platonova & Potin, 1972 and Ethmolaiminae Filipjev & Schuurmans Stekhoven, 1941 (Chromadorida) and family Onchulidae Andrássy, 1963 (Triplonchida) (Holovachov et al., 2008) (Fig. 8). Such remarkable resemblance in structure of mouth apparatus may be explained by convergence caused by similarity in feeding behavior. Unusual for enoplids spiral amphids has also been described for representatives of the family Enchelidiidae Filipjev, 1918 (for example *Aronema* Fadeeva & Belogurov, 1988, *Bernardius* Da Fonseca-Genevois et al., 2009, *Belbolla* (Cobb, 1920) Andrássy, 1973, *Ditlevsenella* Filipjev, 1927, *Eurystomina* Filipjev, 1921). Until now representatives of the family Ironidae were characterized by the cup-shaped *fovea amphidialis* or shape of amphid was not mentioned at all. So, the presence of unispiral amphideal fovea described for the first time for the family Ironidae.

Another remarkable feature of the new species is the presence postcloacal tubular organs. These structures resemble lateral accessory pieces found in many Enopleans, such as *Thrissonchulus provulvatus* Orcelly & Vincoguerra, 1997. After detailed study of these structures we found out that they had no connection with spicules or each other and have its own opening (Fig. 6E). Unfortunately, the shortage of material does not allow as conducting additional investigation in order to enlighten the nature and origin of these structures. We suppose that they are functioning as postcloacal supplementary organs.

Postcloacal supplementary organs have been described in different families of nematodes such as Linhomoeidae Filipjev, 1922 (*Linhomoeus caudipapillosus* Gollner, Miljutina & Bright, 2013), Desmodoridae Filipjev, 1922 (*Parabostrichus bathyalis* Tchesunov, Ingels & Popova, 2012; *Desmodorella schulzi* (Gerlach, 1950)), Trichodoridae Thorne, 1935. However, in all cases postcloacal supplements have been described as papillae. The presence of the tubular supplement is characteristic of the species of the genus *Ironella*, but they are located anterior to cloaca. The presence of pair of tubular postcloacal organs described for *Piipironus grandis* gen. et sp. nov. is, as far as we know, unique among nematodes.

## Abbreviations

a: body length divided by maximum body diameter
a.b.d.: anal body diameter (μm)
amph. dist.: distance from anterior end to amphid (µm)
amph.W.: width of the amphideal fovea (μm)
b: body length divided by pharyngeal length
c’: tail length divided by corresponding body diameter at cloacal level
c: body length divided by tail length
diam.c.s.: body diameter at the level of cephalic setae (μm)
gub. L.: length of gubernaculum (μm)
L: body length (μm)
l.c.s.: length of cephalic setae (μm)
l.tail: tail length (μm)
M: maximum body diameter (μm)
o.l.s.: outer labial setae (µm)
ph. L: pharyngeal length (μm)
ph.b.d.: body diameter at the level of caridia (μm)
S’: length of spicules divided by a.b.d.
spic. Arch: length of spicule along the arch (μm)
V: distance of the vulva from the anterior end (μm)
V (%): distance of the vulva from the anterior end as percentage of body length (%)

## Additional Information and Declarations

### Data Availability

The following information was supplied regarding data availability: The raw measurements are available in the Supplemental File. All specimens are deposited in the Museum of A.V. Zhirmunsky National Scientific Center of Marine Biology FEB RAS (Vladivostok, Russia). Holotype male slide MIMB 42849 Paratype male 1 slide MIMB 42850 Paratype male 2 slide MIMB 42850 Paratype male 3 slide MIMB 42851 Paratype male 4 slide MIMB 42851 Paratype female 1 slide MIMB 42852 Paratype female 2 slide MIMB 42852

### New Species Registration

The following information was supplied regarding the registration of a newly described species: Publication LSID: urn:lsid:zoobank.org:pub:FE5905E2-0338-4177-836C-2E9A311F3F0A Piipironus LSID: urn:lsid:zoobank.org:act:63E896E5-3A53-42F5-8321-6B842FF4CED1 *Piipironus grandis* LSID: urn:lsid:zoobank.org:act:A915931B-BF7D-4951-AD72-DA2F3ED8A481
